# Cardiac Microvascular Barrier Function Mediates the Protection of Tongxinluo against Myocardial Ischemia/Reperfusion Injury

**DOI:** 10.1371/journal.pone.0119846

**Published:** 2015-03-17

**Authors:** Kang Qi, Lujin Li, Xiangdong Li, Jinglin Zhao, Yang Wang, Shijie You, Fenghuan Hu, Haitao Zhang, Yutong Cheng, Sheng Kang, Hehe Cui, Lian Duan, Chen Jin, Qingshan Zheng, Yuejin Yang

**Affiliations:** 1 State Key Laboratory of Cardiovascular Disease, Fuwai Hospital, National Center for Cardiovascular Disease, Chinese Academy of Medical Sciences and Peking Union Medical College, Beijing, 100037, China; 2 Center for Drug Clinical Research, Shanghai University of Chinese Medicine, Shanghai, 201203, China; 3 Biological Information and Statistics Center, Fuwai Hospital, National Center for Cardiovascular Disease, Chinese Academy of Medical Sciences and Peking Union Medical College, Beijing, 100037, China; Indiana University School of Medicine, UNITED STATES

## Abstract

**Objective:**

Tongxinluo (TXL) has been shown to decrease myocardial necrosis after ischemia/reperfusion (I/R) by simulating ischemia preconditioning (IPC). However, the core mechanism of TXL remains unclear. This study was designed to investigate the key targets of TXL against I/R injury (IRI) among the cardiac structure-function network.

**Materials and Methods:**

To evaluate the severity of lethal IRI, a mathematical model was established according to the relationship between myocardial no-reflow size and necrosis size. A total of 168 mini-swine were employed in myocardial I/R experiment. IRI severity among different interventions was compared and IPC and CCB groups were identified as the mildest and severest groups, respectively. Principal component analysis was applied to further determine 9 key targets of IPC in cardioprotection. Then, the key targets of TXL in cardioprotection were confirmed.

**Results:**

Necrosis size and no-reflow size fit well with the Sigmoid Emax model. Necrosis reduction space (NRS) positively correlates with I/R injury severity and necrosis size (*R^2^*=0.92, *R^2^*=0.57, *P*<0.01, respectively). Functional and structural indices correlate positively with NRS (*R^2^*=0.64, *R^2^*=0.62, *P*<0.01, respectively). TXL recovers SUR2, iNOS activity, eNOS activity, VE-cadherin, β-catenin, γ-catenin and P-selectin with a trend toward the sham group. Moreover, TXL increases PKA activity and eNOS expression with a trend away from the sham group. Among the above nine indices, eNOS activity, eNOS, VE-cadherin, β-catenin and γ-catenin expression were significantly up-regulated by TXL compared with IPC (P>0.05) or CCB (P<0.05) and these five microvascular barrier-related indices may be the key targets of TXL in minimizing IRI.

**Conclusions:**

Our study underlines the lethal IRI as one of the causes of myocardial necrosis. Pretreatment with TXL ameliorates myocardial IRI through promoting cardiac microvascular endothelial barrier function by simulating IPC.

## Introduction

Tongxinluo (TXL) is extracted and concentrated from a group of herbal medicine, such as ginseng, radix paeoniae rubra, borneol, and spiny jujuba seed [1]. TXL was registered in the State Food and Drug Administration of China for treatment of angina pectoris in 1996. Recent studies confirmed that as a stable formulation, TXL capsule could stabilize coronary plaque [2] and reduce myocardial necrosis size with few adverse effects [3]. Therefore in China TXL has been extensively used for the treatment of patients with any clinical type of coronary artery disease, including acute coronary syndrome.

Previous studies confirmed several drugs including TXL could rescue a large extent of myocardium through prevention of no-reflow (Table 1). Our previous studies have shown that pretreatment with TXL at low loading dose 1-h before ischemia protects reperfused hearts by enhancement of endothelial nitric oxide (NO) synthase (eNOS) activity and NO bioavailability while inhibiting myocardial inflammation, apoptosis, and edema via protein kinase A (PKA) pathway[4,5]. Recent studies showed that beneficial cardiac effects of ginseng (one of constituents of TXL) related to attenuation of calcineurin activation via sodium-hydrogen exchanger isoform-1 inhibition [6,7]. However, lethal ischemia/reperfusion (I/R) injury (IRI), another cause of myocardial necrosis, has not been evaluated under TXL intervention. Moreover, the entirety of the cardioprotective effects of TXL cannot be explained by a single mechanism because of its complicated composition and possible pleiotropic effects. Therefore, the key target of TXL against IRI needs to be clarified in terms of clinical benefits for patients with AMI. In this study, we hypothesized that key target from cardiac structure and (or) cardiac function mediated the protection of TXL against IRI.

**Table 1:** Data sources for mathematical modeling.

	Age(month)	Sex (Male/female)	BW(kg)	Anesthesia	MAP(mmHg)	HR (beats/min)	Mechanical ventilation	Ligation site	Ligation time	Reperfusion time	Model group(No.)	IPC group(No.)
Zhao JL, 2005[8]	6	12/12	30±3	10 mg/kg of azaperone, 10 mg/kg of thiopental	84.3±12.1	105.3±6.6	Yes	LAD	3-h	1-h	8	8
Zhao JL, et al, 2006[9]	6	31/31	30.3±3	10 mg/kg of azaperone, 10 mg/kg of thiopental	83.8±9.5	103.8±3.5	Yes	LAD	3-h	2-h	10	0
Yang YJ, et al, 2006[10]	6	20/20	30.3±3	10 mg/kg of azaperone, 10 mg/kg of thiopental	86.0±9.0	109.0 ± 5.0	Yes	LAD	3-h	1-h	8	8
Zhao JL, et al, 2006[11]	6	31/31	30±3	10 mg/kg of azaperone, 10 mg/kg of thiopental	85.0±11.0	108.0±6.0	Yes	LAD	3-h	2-h	9	0
Zhao JL, et al, 2006[12]	6	28/28	30±3	10 mg/kg of azaperone, 10 mg/kg of thiopental	85.0±10.0	109.0 ± 5.0	Yes	LAD	3-h	2-h	8	0
Zhao JL, et al, 2006[13]	6	22/22	30.3±3	10 mg/kg of azaperone, 10 mg/kg of thiopental	86.0±8.0	94.0±5.0	Yes	LAD	3-h	1-h	10	0
Zhao JL, et al, 2007[14]	6	12/12	30.3±3	10 mg/kg of azaperone, 10 mg/kg of thiopental	86.0±9.0	109.0 ± 5.0	Yes	LAD	3-h	2-h	8	0
Zhao J, et al, 2007[15]	6	16/16	30.3±3	10 mg/kg of azaperone, 10 mg/kg of thiopental	88.0±10.0	90.0±5.0	Yes	LAD	3-h	1-h	8	0
Yang YJ, et al, 2007[16]	6	23/22	30±3	10 mg/kg of azaperone, 10 mg/kg of thiopental	82.5±10.1	98.3±6.3	Yes	LAD	3-h	4-w	10	0
Cheng YT, et al, 2009[17]	6	14/14	25±3	ketamine hydrochloride 700 mg, diazepam 30 mg	83.9±11.6	96.20±3.12	Yes	LAD	90-min	3-h	7	0
Cheng YT, et al, 2009[18]	6	10/9	30±3	ketamine hydrochloride 700 mg, diazepam 30 mg	84.1±11.4	96.2 ±3.1	Yes	LAD	90-min	3-h	7	0
Zhang HT, et al, 2009[19]	6	20/20	25±3	ketamine hydrochloride 700 mg, diazepam 30 mg	80.4±13.1	102.3±7.6	Yes	LAD	90-min	3-h	8	0
Duan L, et al, 2010[20]	6	15/15	30±3	ketamine hydrochloride 700 mg, diazepam 30 mg	83.5±10.2	95.1 ±2.7	Yes	LAD	3-h	1-h	6	0
Li XD, et al, 2010[4]	6	20/20	20–30	ketamine hydrochloride 700 mg, diazepam 30 mg	84.56±10.21	102.63±10.34	Yes	LAD	90-min	3-h	8	0
Duan L, et al, 2010[21]	6	15/15	30±3	ketamine hydrochloride 700 mg, diazepam 30 mg	85.3±12.8	101.7±9.4	Yes	LAD	3-h	1-h	6	0
Li XD, et al, 2012[22]	6	12/12	20–30	ketamine hydrochloride 700 mg, diazepam 30 mg	88.3±11.5	98.6±7.8	Yes	LAD	90-min	3-h	6	0
Li XD, et al, 2012[23]	6	24/24	20–30	ketamine hydrochloride 700 mg, diazepam 30 mg	83.4 ±11.7	97.9 ±10.6	Yes	LAD	90-min	3-h	7	7
Li XD, et al, 2012[24]	6	27/27	20–30	ketamine hydrochloride 700 mg, diazepam 30 mg	84.6±10.2	102.6±10.3	Yes	LAD	90-min	3-h	8	0
Li XD, et al, 2013[25]	6	18/17	20–30	ketamine hydrochloride 700 mg, diazepam 30 mg	80.3±12.1	93.1±11	Yes	LAD	90-min	3-h	7	0

Abbreviations:

BW = body weight;

MAP = mean aortic pressure;

HR = heart rates;

IPC = ischemia preconditioning.

## Material and Methods

### 2.1 Data sources

**Fig 1:**
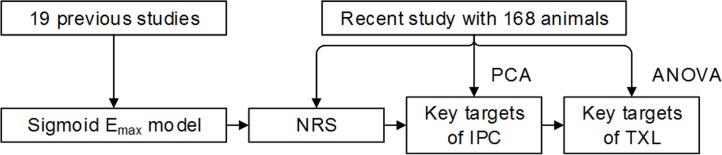
Data sources. Data from our 19 previous studies were used for mathematical modeling only. Data from our present experiment were used for calculating NRS and investigating mechanism of IPC and TXL. Abbreviations: NRS = necrosis reduction space; IPC = ischemia preconditioning; PCA = principal component analysis.

The data in this study had two sources (Fig. 1). Our 19 previous animal studies offered the data for mathematical modeling only. And data from our present experiment with 168 animals were used for lethal IRI severity evaluation, principal component analysis (PCA) and consequent mechanism investigation of TXL.

### 2.2 Mathematical modeling

To uncover the relationship between myocardial no-reflow size and necrosis size, mathematical modeling was employed. All data used for mathematical modeling were obtained from 19 myocardial I/R mini-swine studies in Fuwai Hospital (Table 1). All the animals were bought from fine-breed breeding farm, Peking Agricultural University. The surgery of all animals was performed in Animal Experiment Center of Fuwai hospital and the crucial surgery processes (i.e. ligation, reperfusion and injection) were accomplished by the same experienced surgeon. Animals in ischemia preconditioning groups (IPC, n = 23) and model groups (n = 149) were free from drug treatment, so necrosis sizes and no-reflow sizes from the two groups were presented in a scatter plot to demonstrate the relationship between necrosis size and no-reflow size during natural disease process. Different I/R time in these studies caused different necrosis sizes and no-reflow sizes, thus expanding data distribution and model application. Therefore, the diversity in I/R time could reflect the actual relationship between necrosis size and no-reflow size.

**Fig 2:**
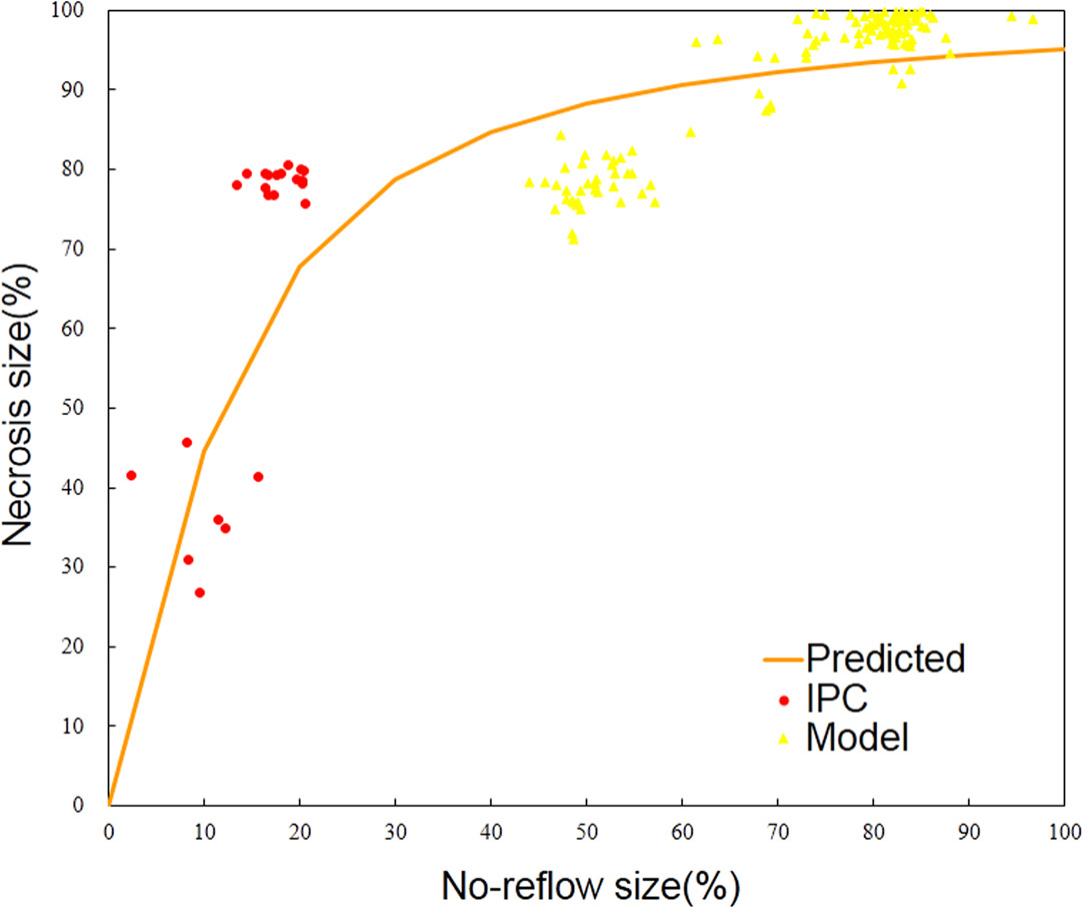
Relationship between no-reflow size and necrosis size. Myocardial necrosis size and no-reflow size fit a Sigmoid E_max_ model well. Myocardial necrosis size enlarges with the increase of no-reflow size. Abbreviations: IPC = ischemia preconditioning.

Sigmoid E_max_ is the classic model in pharmacodynamics, demonstrating the dose-effect relationship. Interestingly, our study showed that scatter points of no-reflow size (dose) and necrosis size (effect) fit well in a Sigmoid E_max_ model (Fig. 2, Formula 1). When the no-reflow size is 0%, the necrosis size is 0%. As the no-reflow size increases, the necrosis size elevates. When the no-reflow size continues to increase, the increase of necrosis size slows down, i.e. the necrosis size approaches a plateau. When the no-reflow size is 100%, the necrosis size is 100%. The model was selected according to the distribution character of data. Model parameters were obtained by the NONMEM method (S1 Table). E_AN_ is the estimate of necrosis size. E_ANR_ is the observational no-reflow size. E_max_ is the largest necrosis size (E_max_ = 100%). EC_50_ is the no-reflow size when estimate of necrosis size is equal to 50% (E_AN_ = 50%). r is the slope factor. ISV is the variation among trials. ε is the individual residual.

$$E_{AN}\;=\;\frac{E_{max}*E_{ANR}^{r}}{{EC}_{50}+E_{ANR}^{r}}+ISV+\varepsilon$$ (Formula 1)

To confirm the accuracy of the model, the bias was analyzed as shown in S1 Fig. A-D. The predictive ability of this model was validated as shown in S1 Fig. E. Accordingly, in our study both ischemia time and reperfusion time have no significant effect on the relationship between necrosis size and no-reflow size. The estimates of necrosis sizes could be accurately quantified based on this model.

### 2.3 Animal experiment protocol

**Fig 3:**
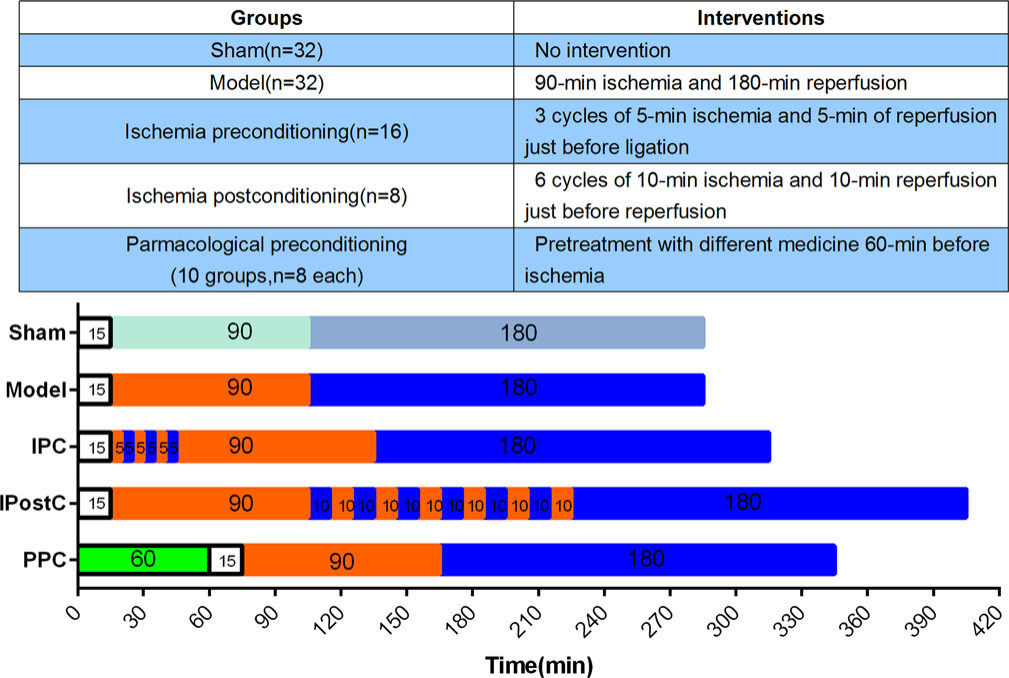
Animal Experiment protocol. Pharmacological preconditioning includes TXL, valsartan, nicorandil, rosuvastatin, simvastatin, carvedilol, tirofiban, diltiazem, verapamil and adenosine. Blood collection and measurement of functional parameters were done at before ligation, before reperfusion and before sacrifice. No-reflow and necrosis sizes were obtained after sacrifice. Abbreviations: IPostC = ischemia postconditioning; IPC = ischemia preconditioning; PPC = pharmacological preconditioning.

Bought from fine-breed breeding farm, Peking Agricultural University, a total of 168 sex-matched Chinese mini-swine weighing 25±5 kg were employed. All animals used in this study received humane care in compliance with the National Institutes of Health *Guide for the Care and Use of Laboratory Animals*. The protocol was approved by the Committee on the Ethics of Animal Experiments of the Fuwai Hospital (Permit Number: 2013–4/5–100/30–973). The surgery was performed in Animal Experiment Center of Fuwai hospital and the crucial surgery processes were accomplished by the same experienced surgeon. All surgery was performed under anesthesia with a mixture of ketamine hydrochloride 700 mg and diazepam 30 mg intramuscularly and were continuously infused with the mixture (2 mg·kg^−1^·h^−1^) intravenously, and all efforts were made to minimize suffering. Animals were randomly assigned to sham, model or different intervention groups. The mini-swine underwent a 90-min of ligation and 3-h of reperfusion of the left anterior descending (LAD) coronary artery, with the following interventions (Fig. 3):

(1) Sham (n = 32): the LAD was only encircled by a suture without occlusion;
(2) Model (n = 16): no intervention either before or after LAD occlusion;
(3) IPC (n = 16): 3 cycles of 5-min of ischemia and 5-min of reperfusion before the prolonged ischemia [23];
(4) Ischemia postconditioning (IPostC) (n = 8): 6 cycles of 10-min of ischemia and 10-min of reperfusion before the prolonged reperfusion;
(5) Pharmacological preconditioning(10 groups, n = 8 each): doses based on clinical usage in acute coronary syndrome and previous animal studies, with the following medicines intragastrically (i.g.) administered 1-h before LAD ligation: TXL(50 mg/kg, Yiling Co., Ltd., China)[4], valsartan(2 mg/kg, Novartis Co., Ltd., USA), rosuvastatin (4 mg/kg, AstraZeneca Co., Ltd., China), simvastatin (2 mg/kg, Merck Co., Ltd., USA) [24,25] and carvedilol (1 mg/kg, Roche Co., Ltd., Switzerland). Tirofiban (Yuanda Co., Ltd., China) was given as a 15 μg/kg intravenous (i.v.) bolus, followed by an infusion administered at a rate of 0.3 μg/kg/min from 1-h before ligation to the end of protocol. Nicorandil (Libang Co., Ltd., China) was administered as a 120 μg/kg i.v. bolus, followed by an infusion administered at a rate of 4 μg/kg/min from 1-h before ligation to the end of protocol. Adenosine (Everbright Co., Ltd., China) was administered as an i.v. infusion at a rate of 100 μg/kg/min from 1-h before ligation to the end of protocol. Diltiazem (Tanabe Seiyaku Co., Ltd., Osaka) and verapamil (Vasolan Eisai Co., Ltd., Tokyo) were administered as an intracoronary bolus of 2 mg immediately before reperfusion within 1-min.

Blood samples were drawn before sacrifice. Then no-reflow and necrosis size was measured immediately after sacrifice. Next, myocardial tissue samples were harvested.

### 2.4 Hemodynamic parameters determination

Hemodynamic parameters were measured at baseline, after 90-min of ischemia, and after 3-h of reperfusion, including heart rate (HR), mean aortic pressure, left ventricular end-diastolic pressure (LVEDP), maximum and minimum rates of left ventricular pressure development (d*P*/d*t*_max_ and d*P*/d*t*_min_).

### 2.5 Measurement of necrosis size and no-reflow size

Necrosis and no-reflow sizes were measured among model (n = 32), IPC (n = 16), IPostC (n = 8) and pharmacological preconditioning (10 groups, n = 8 each) groups as previously described [25]. Briefly, at the end of reperfusion, 4% thioflavin S (1 ml/kg, Sigma, USA) was injected into the left atrium to delineate the area of no-reflow. LAD was re-ligated within 30-min, and then 2% Evans blue dye (1 ml/kg, Sigma, USA) was injected into the left atrium to outline the area at risk, indicating I/R territory of LAD. The deeply anesthetized swine was sacrificed by an injection of 15% KCl (1 ml/kg). Then the heart was excised and rinsed in ice-cold saline solution to remove the blood and excess dye. The atria and right ventricular free wall were removed, and the remaining left ventricular tissue was sectioned perpendicular to its long axis into six to seven sections and photographed. Area at risk, the area unstained by Evans blue, was traced and pictured in visible light. Area of no-reflow, the area not perfused by thioflavin S, was photographed using ultraviolet light (wavelength: 365 nm) and a yellow filter. The area between the area at risk and area of no-reflow was the area of reflow. Then tissue samples were collected immediately from the area of reflow and were placed in liquid nitrogen for further study. Finally, tissue slices were weighed and incubated in 1% triphenyltetrazolium chloride (TTC) at 37°C for 15-min. TTC stains viable myocardium red, and necrotic tissue appears pale. After computerized planimetry, the percentage of the area was multiplied by the slice weight and then accumulated for different myocardial slices. Outcomes were calculated as follows: Size at risk(%) = (mass at risk/mass of left ventricle)×100%; No-reflow size(%) = (mass of no-reflow /size at risk)×100%; Necrosis size(%) = (mass of necrosis area/size at risk)×100%.

### 2.6 Lethal IRI severity quantification

**Fig 4:**
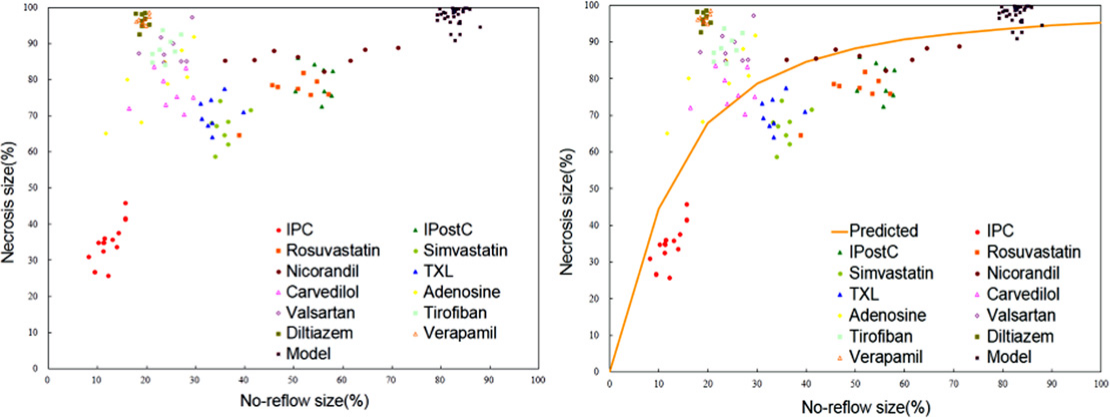
Scatterplot for observational values of necrosis and no-reflow sizes in the model group and all intervention groups. **A**: Scatterplot for necrosis sizes and no-reflow sizes; **B**: Scatterplot and established model. Under similar no-reflow sizes, differences in necrosis size existed with different interventions. Diltiazem and verapamil reduce no-reflow size and necrosis size to a similar degree. IPC, verapamil and diltiazem reduced no-reflow size to a similar degree, but IPC caused a greater decrease in necrosis size than diltiazem or verapamil.

**Fig 5:**
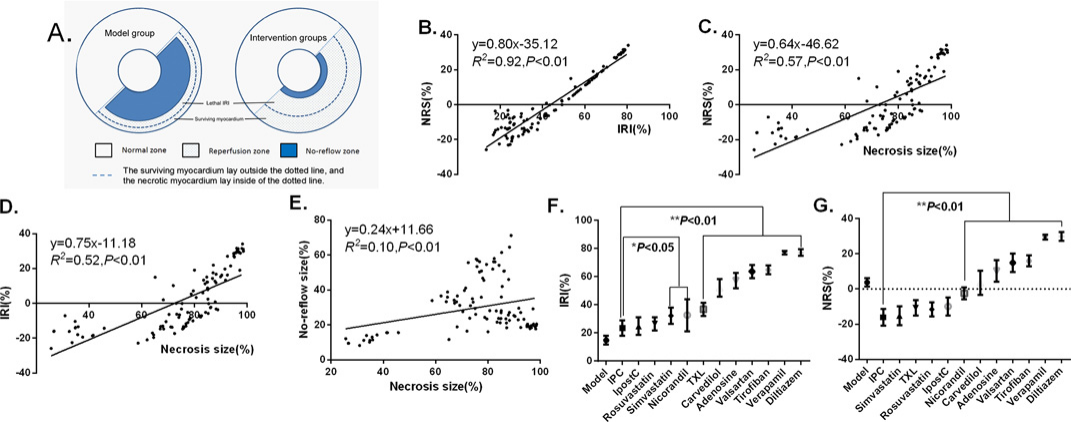
Comparison of lethal IRI under all interventions. **A**: Illustration for lethal IRI zone. The no-reflow zone was unstained using thioflavin S, and the necrotic zone was unstained using TTC. The surviving myocardium lies outside the dotted line, and the necrotic myocardium lies inside of the dotted line. **B**: The positive correlation between NRS value and IRI value (*R* ^*2*^ = 0.92, P<0.01) indicates NRS is a valuable surrogate marker for lethal IRI severity. **C-D**: Necrosis size is positively correlated with NRS (*R* ^*2*^ = 0.57, P<0.01) or IRI value (*R* ^*2*^ = 0.52, P<0.01), underlining the linear relationship between IRI and necrosis size. **E**: Necrosis size has weak linear relationship with no-reflow size (*R* ^*2*^ = 0.10, P<0.01). **F-G**: Observational values of NRS and IRI were expressed as the mean±SE; the error bars were the standard deviations. The significance of differences between groups was analyzed using the one way ANOVA, *P<0.05, **P< 0.01 vs IPC group. Abbreviations: NRS = necrosis reduction space; IRI = ischemia/reperfusion injury.

To quantitatively compare the effects of different interventions on the severity of lethal IRI, observational values of necrosis size and no-reflow size from all intervention groups were displayed in Fig. 4A, B. Lethal IRI severity was evaluated by pathological staining with or without mathematical modeling respectively. In the model-based pathological approach, NRS was introduced and calculated as follows: NRS = observational necrosis size—estimated necrosis size. Alternatively, in the simple pathological approach, lethal IRI was calculated as previously described [26], with modification: IRI = observational necrosis size—observational no-reflow size (Fig. 5A). Then the association between NRS and IRI was tested by Spearman correlation analysis.

### 2.7 Laboratory analyses

With the highest and the lowest NRS respectively, calcium channel blockers (CCB) and IPC groups were chosen for laboratory analysis. Meanwhile, TXL group, purpose of this study, and drug-untreated model group were also selected for laboratory analysis. To reveal the full picture of cardiac structure-function networks, 24 markers were measured, such as VE-cadherin, eNOS activity etc. in the Center Laboratory of Fuwai Hospital (Table 2, S1 Materials and Methods). Data from sham (n = 32), model (n = 32), IPC (n = 16), verapamil (n = 8), diltiazem (n = 8) and TXL (n = 8) groups were compared.

**Table 2:** Cardiac structure-function network indices.

Categories	Subcategories	Indices
Functional indices	K_ATP_ function	SUR2, Kir6.1, Kir6.2;
	Enzyme activity	PKA activity, eNOS activity, iNOS activity;
	Endothelial function	eNOS, Ser^1179^p-eNOS, Ser^635^p-eNOS;
Structural indices	Endothelial junction	VE-cadherin, β-catenin, γ-catenin;
	Neutrophil migration	P-selectin, ICAM-1, VCAM-1, Neutrophil infiltration;
	Water transportation	Water content, CSA, MSA, FITC concentration, AQP-1, AQP-4, AQP-8, AQP-9.

Laboratory data include adenosine triphosphate-sensitive K^+^(K_ATP_) channel proteins SUR2, Kir6.1 and Kir6.2, protein kinase A(PKA) activity, eNOS activity, iNOS activity, eNOS, Ser^1179^p-eNOS, Ser^635^p-eNOS, VE-cadherin, β-catenin, γ-catenin, P-selectin, intercellular adhesion molecule-1 (ICAM-1), vascular cell adhesion molecule-1(VCAM-1), neutrophil infiltration, myocardial water content, cardiomyocyte cross-sectional area (CSA), mitochondria cross-sectional area (MSA), myocardial FITC concentration and aquaporin-1, -4, -8 and -9 (AQP-1, 4, 8, 9).

The expression of eNOS, Ser^1179^p-eNOS, Ser^635^p-eNOS, SUR2, Kir6.1 and Kir6.2, VE-cadherin, β-catenin, γ-catenin, AQP-1, -4, -8, and -9 in the area of reflow was detected using Western blot. Rabbit polyclonal eNOS (Cell Signaling, USA), rabbit monoclonal p-eNOS (Ser^1179^, Invitrogen, USA), rabbit polyclonal p-eNOS (Ser^635^, Upstate, USA), goat polyclonal SUR2, Kir6.1 and Kir6.2 (Santa Cruz, USA), goat polyclonal VE-cadherin (Santa Cruz, USA), mouse monoclonal β-catenin and γ-catenin (Transduction Labs, USA), rabbit polyclonal AQP-1 (Abcam, UK), rabbit polyclonal AQP-4 (Abcam, UK), rabbit polyclonal AQP-8 (Santa Cruz, USA), goat polyclonal AQP-9 (Santa Cruz, USA), or mouse monoclonal β-actin (Proteintech group, USA) antibodies were applied. As previously described, myocardial PKA activity was determined using a nonradioactive PKA assay kit (Promega, USA) [4,25,27]. Myocardial activities of iNOS, and cNOS were measured using a spectrophotometrical assay kit (Nanjing JianCheng, China) [17,23]. Plasma P-selectin, ICAM-1, VCAM-1, markers of neutrophil-mediated inflammation, were measured using Elisa kits (Rapidbio, USA) after 3-h of reperfusion according to the manufacturer’s instructions.

Neutrophil infiltration in the area of reflow was semi-quantified by light microscopy (Nikon eclipse E400, Japan) at 400× magnifications in a blinded manner by a cardiac pathologist, as previously described [17]. To quantify mitochondrial edema, mitochondria cross-sectional area (MSA) was measured at a magnification of 30,000× with a JEOL JEM-1230 transmission electron microscope.

LV and tissue water content was calculated as water content (%) = [(wet weight−dry weight)/ wet weight] ×100%, as reported previously [25]. Myocardial FITC concentration was measured using FITC-dextran (70 kDa, Sigma, USA) and was later quantified using a fluorescence spectrophotometer as previously described [25,28].

### 2.8 Principal component analysis

To determine whether key target from cardiac structure and (or) cardiac function mediates the protection of TXL against lethal IRI, PCA was employed. IPC is the gold standard against which all experimental cardioprotective agents are judged [29]. CCB has been widely used in patients with coronary artery disease. With the highest and the lowest NRS respectively, IPC and CCB groups were chosen for PCA. Discriminating indices from cardiac structure and (or) function may explain cardioprotection of IPC and TXL. To test this hypothesis, these indices were quantitatively compared among model, TXL, IPC and CCB groups.

To test the association between NRS and multi-marker index of cardiac structure or multi-marker index of cardiac function, Spearman correlation analysis was applied.

### 2.9 Data processing

The data for mathematical modeling were analyzed using NONMEM 7.1.0 software (ICON Development Solutions, USA). Laboratory data were expressed as the mean±SD and the difference among groups was compared by one-way ANOVA. All data processing and PCA were performed using R 3.0.1 software. The association between two markers was analyzed by Spearman correlation analysis. Statistical significance was set at *P*<0.05.

## Results

### 3.1 The relationship between no-reflow size and necrosis size

In our study, myocardial necrosis size would be enlarged with the increase of no-reflow size. The relationship between no-reflow size and necrosis size fits the Sigmoid E_max_ model (Fig. 2). When the no-reflow size reaches 11.7%, the necrosis size is 50% (EC_50_ is 11.7%). The relative standard deviation is less than 20%, suggesting parameter variation is small enough to ensure the reliability of the parameter estimates (S1 Table).

Estimated necrosis size could be accurately predicted with known no-reflow size. Biological evidences were provided in model diagnostic figure by comparing the observational necrosis sizes and estimated necrosis sizes (S1 Fig.). Both population and individual estimates are associated with observational values and the trend line is close to diagonal (S1 Fig. A, B). Furthermore, the weight relative standard deviations are almost evenly distributed between-4 and 4 (S1 Fig. C, D), indicating good predictive capacity of this model. Observational necrosis sizes are almost evenly dispersed in the predicted 90% (5% and 95% quantile) confidence intervals (CI) and on both sides of the median lines, suggesting a good capacity of this model to predict the necrosis size (S1 Fig. E). Distribution of observational values of necrosis and no-reflow sizes in different groups was shown in S2 Fig., indicating this model could also be applied under different drug treatments.

### 3.2 Comparison of hemodynamics under different interventions

In animal study, ventricular fibrillation occurred in 1 mini-swine in model group 30-min after reperfusion but was converted to sinus rhythm by an intrathoracic defibrillation at 10 J. Further experiment was proceeded after stabilization. Although there were no differences in cardiac hemodynamics among all groups at baseline (*P*>0.05), HR, LVEDP and d*P*/d*t*_max_ were increased and d*P*/d*t*_min_ were decreased in the untreated model groups (*P*<0.05), which were partially ameliorated by all intervention groups (Table 3).

**Table 3:** Hemodynamic data at baseline, at the end of ischemia and at the end of reperfusion.

	HR (beats/min)	MAP (mmHg)	LVEDP (mmHg)	+d*P*/d*t*_max_	-d*P*/d*t*_min_ (mmHg/s)
**Baseline**					
Sham	99.5±3.2	97.0±7.7	5.0±0.8	1998.1±152.3	-1563.3±198.4
Model	98.3±6.1	99.3±10.3	5.7±0.9	1874.2±145.2	-1545.3±173.4
IPC	105.2±8.2	100.7±11.4	4.6±1.6	1957.3±165.3	-1573.5±204.5
IPostC	97.8±7.6	110.6±9.1	5.4±0.5	1953.2±163.3	-1497.2±187.4
Simvastatin	102.5±6.2	93.8±7.9	5.2±0.7	1898.4±174.1	-1449.3±202.4
TXL	103.3±7.0	95.2±6.3	4.7±1.3	1892.4±168.3	-1584.2±167.3
Rosuvastatin	98.3±9.8	92.1±6.3	5.7±1.2	1956.1±167.4	-1493.2±205.3
Nicorandil	106.4±10.5	102.5±7.1	5.2±1.0	1982.2±188.4	-1502.1±178.0
Carvedilol	104.2±8.1	100.4±6.9	5.5±0.9	1936.2±166.2	-1498.3±214.2
Adenosine	98.5±6.2	95.8±12.4	5.4±0.7	2017.4±164.1	-1559.3±179.4
Valsartan	99.3±7.0	107.5±16.1	5.4±0.5	2011.4±168.3	-1489.2±159.5
Tirofiban	98.3±9.3	105.4±3.0	5.2±1.0	1973.1±177.4	-1524.5±149.5
Verapamil	96.2±7.5	96.7±10.2	4.8±0.6	2006.2±158.4	-1593.5±231.4
Diltiazem	101.2±8.0	94.5±9.6	5.9±1.3	1935.2±166.2	-1496.3±185.9
**Ischemia 90-min**					
Sham	90.5±2.9	99.4±10.0	4.9±1.0	1901.1±150.5	-1294.3±130.5
Model	108.3±4.7**	89.9±7.5	4.9±1.3	1434.2±141.1**	-739.3±87.3**
IPC	103.2±8.6**	92.4±9.0	6.1±1.0^△^	1857.3±163.2^##^	-1104.4±119.2* ^##^
IPostC	86.8±4.5^##^ ^△△^	93.9±8.1	4.5±0.9	1852.5±143.1^##^	-1038.2±95.8** ^##^
Simvastatin	80.5±5.6* ^##^ ^△△^	92.2±7.7	5.8±0.8	1814.1±164.1^##^	-1204.4±104.8^##^
TXL	90.3±6.0^##^ ^△△^	79.3±4.3**	5.1±1.0	1810.4±137.3^##^	-1285.3±157.3^##^ ^△△^
Rosuvastatin	85.7±7.9^##^ ^△△^	87.6±8.9	5.7±0.9	1821.1±127.4^##^	-1284.3±179.2^##^ ^△^
Nicorandil	90.4±8.5^##^ ^△△^	77.5±11.0**	5.0±0.8	1782.2±129^##^	-1004.3±98.3** ^##^
Carvedilol	99.2±8.7	98.3±8.6	5.2±0.3	1826±146.2^##^	-1084.4±130.3** ^##^
Adenosine	83.5±4.8^##^ ^△△^	90.4±11.2	5.5±1.0	1817.3±134.1^##^	-980.4±128.2** ^##^ ^△^
Valsartan	85.3±5.7^##^ ^△△^	99.5±8.1	5.6±0.7	1913.3±148.3^##^	-1038.4±98.4** ^##^
Tirofiban	90.2±7.4^##^ ^△△^	86.9±7.9	5.1±0.8	1935.1±127.4^##^	-989.4±104.5** ^##^
Verapamil	87.1±4.4^##^ ^△△^	89.4±5.3	4.8±0.6	1896.2±131.4^##^	-1058.3±89.4** ^##^
Diltiazem	91.2±5.0^##^ ^△△^	96.7±11.1	5.2±1.0	1902.2±146.2^##^	-1085.3±88.4** ^##^
**Reperfusion 180-min**					
Sham	74.5±6.2	69.4±7.1	4.7±0.7	1878.1±152.7	-963.2±87.5
Model	76.3±6.4	61.8±5.6	5.2±1.3	1234.2±145.2**	-624.2±99.3**
IPC	79.2±8.8	61.0±7.8	5.7±0.9	1757.3±153.6^##^	-958.2±103.3^##^
IPostC	80.8±7.4	62.2±5.8	5.5±0.7	1653.2±153.3* ^##^	-1049.2±132.4^##^ ^△△^
Simvastatin	86.5±4.8	66.0±5.2	6.0±0.9	1614.2±164.4* ^##^	-1049.3±89.3^##^
TXL	86.3±5.6** ^#^	69.6±9.3	5.3±0.6	1535.6±139.2**	-1035.3±88.3^##^ ^△^
Rosuvastatin	82.3±7.9	62.3±4.4	5.5±0.8	1604.6±129.5** ^##^	-984.2±79.3^##^
Nicorandil	90.4±9.5* ^#^	56.4±5.7	4.7±0.6	1483.5±140.2** ^△^	-947.2±104.5^##^
Carvedilol	87.2±8.4	67.5±8.5	5.5±1.1	1532.1±149.3** ^##^	-1048.5±89.4^##^
Adenosine	92.5±5.2** ^##^	67.2±10.5	5.8±1.0	1817.2±164.8^##^	-984.8±104.2^##^
Valsartan	88.3±4.6	63.7±8.8	5.4±0.7	1791.4±158.2^##^	-894.2±89.3^##^
Tirofiban	83.3±7.0*	60.9±12.1	5.0±1.6	1673.1±187.2^##^	-1034.4±93.5^##^
Verapamil	75.2±7.3	66.4±5.3	4.8±0.5	1825.8±178.2^##^	-943.2±104.5^##^
Diltiazem	81.2±8.4	67.9±12.0	5.9±1.0	1935.2±145.6^##^	-1049.4±89.3^##^ ^△^

Data are means±SE; n = 32 for sham and model groups; n = 16 for IPC group; n = 8 each for the rest groups;

*P<0.05,

**P<0.01 vs Sham,

^#^P<0.05,

^##^P<0.01 vs Model,

^△^P<0.05,

^△△^P<0.01 vs IPC at the same time point.

Abbreviations: IPC = ischemia preconditioning; IPostC = ischemia postconditioning; HR = heart rate; MAP = mean arterial pressure; LVEDP = left ventricular end-diastolic pressure; d*P*/d*t*_max_ and d*P*/d*t*_min_ = maximum and minimum rates of left ventricular pressure development, respectively.

### 3.3 Comparison of lethal IRI severity under different interventions

Lineation of area at risk, area of no-reflow and area of necrosis from model, IPC and TXL groups are shown in S3 Fig. and illustrated by Fig. 5A. Necrosis and no-reflow sizes from different groups are shown in Fig. 4A,B. The necrosis size decreases synchronously with the no-reflow size under most of the cardioprotective drugs included. We used 90% *CI* (5% and 95% quantile) to describe the distribution range of no-reflow size and necrosis size. If the observational values are dispersed in the 90% *CI*, the no-reflow size and necrosis size decrease synchronously; if the observational value is above the 95% *CI* or below the 5% *CI*, the observational value is a small probability event (*P*<0.05), i.e. necrosis size may not decline synchronously with no-reflow size. The observational values for rosuvastatin, simvastatin and TXL are mainly dispersed in the 90% *CI*. However, the observational values for verapamil and diltiazem are above the 95% *CI* (*P*<0.05), suggesting necrosis size may not decline synchronously with no-reflow size under CCB intervention.

The positive correlation between NRS value and IRI value (*R* ^*2*^ = 0.92, *P*<0.01), shown as Fig. 5B, indicates NRS is a valuable surrogate marker for lethal IRI severity. Necrosis size is positively correlated with NRS (*R* ^*2*^ = 0.57, *P*<0.01) or IRI value (*R* ^*2*^ = 0.52, *P*<0.01), underlining the linear relationship between IRI and necrosis size (Fig. 5C,D). However, necrosis size has weak linear relationship with no-reflow size (*R* ^*2*^ = 0.10, *P*<0.01) (Fig. 5E).

Several disadvantages exist for measures of no-reflow and necrosis size simply with pathological staining, such as non-repeatability and low accuracy. Using simple pathological staining, we found lethal IRI is the mildest in IPC among all intervention groups, while is the severest in CCB groups (Fig. 5F). Mathematical modeling could verify the reproducibility of experimental findings and visualize tiny changes accurately and has gained wide adoption in new drug development [30]. As indicated by NRS, pathological staining combining mathematical modeling provides further evidence that lethal IRI is the mildest, followed by simvastatin and TXL, while it is the severest in CCB groups (Fig. 5G). There were similar no-reflow sizes and significantly different necrosis sizes between IPC and CCB groups, suggesting discriminating physiological indices between the IPC and CCB groups may be the core mechanisms of IPC in minimizing lethal IRI and necrosis size. With the highest and the lowest NRS respectively, CCB and IPC groups were chosen for laboratory analyses.

### 3.4 Key targets mediating the protection of TXL

**Fig 6:**
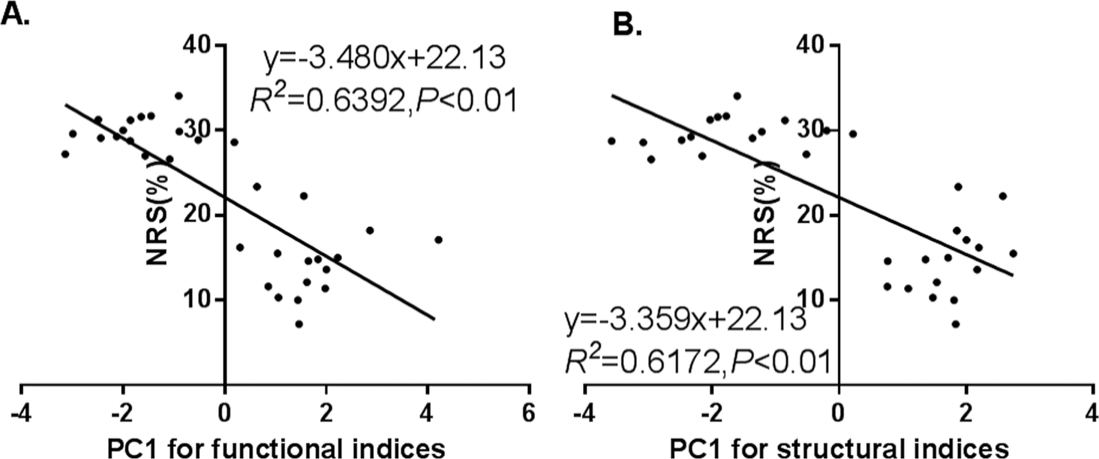
Relationship between NRS and functional or structural indices. **A**: Association between NRS and functional indices. **B**: Association between NRS and structural indices. Abbreviations: NRS = necrosis reduction space; PC1 = Principal Component 1.

To investigate the key targets mediating cardioprotection of TXL, 24 markers of cardiac structure-function network were detected in sham, model, IPC, verapamil, diltiazem and TXL groups. PCA was employed to extract a single weighted multi-marker index of cardiac function from 9 individual indices of cardiac function. The top two principal components (PC1 and PC2) were identified accounting for 54.70% of the explained variance. Meanwhile, a single weighted multi-marker index of cardiac structure was extracted from 15 individual indices. The top two PCs (PC1 and PC2) accounted for 40.57% of the explained variance and no additional significant principal components were identified (Table 4). There was a significant correlation between NRS and PC1 scores for both functional and structural indices (*R* ^*2*^ = 0.64, *R* ^*2*^ = 0.62, *P*<0.01, respectively) (Fig. 6A,B). Therefore, disturbance in both cardiac structure and cardiac function relates to changes of NRS.

**Table 4:** Top two principal component eigenvalues and contribution rates for functional and structural indices.

Categories	No.	Eigenvalue	Percentage(%)	Cumulative Percentage(%)
Functional indices	1	3.68	40.91	40.91
	2	1.24	13.79	54.70
Structural indices	1	3.81	25.44	25.44
	2	2.27	15.12	40.57

Principal component analysis was employed to extract a single weighted multi-marker index of cardiac function from 9 individual indices of cardiac function. The top two principal components (PC1 and PC2) were identified accounting for 54.70% of the explained variance. Meanwhile, a single weighted multi-marker index of cardiac structure was extracted from 15 individual indices. The top two PCs (PC1 and PC2) accounted for 40.57% of the explained variance and no additional significant principal components were identified.

**Fig 7:**
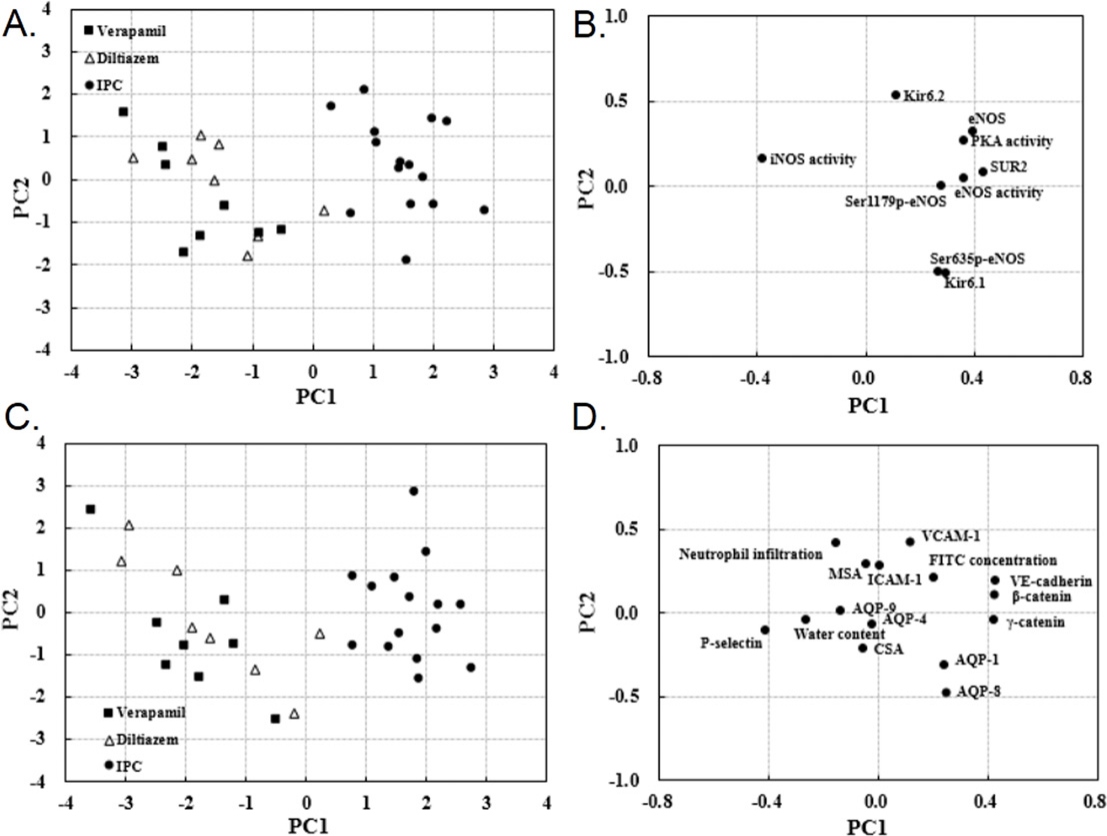
Principal component scatter plot and factor loadings. PCA scores (**A, C**) and loading plots (**B, D**) for the two principal components derived from scores of separate PCA constructed separately for endothelial structure-function network data from the IPC, verapamil and diltiazem groups. **A**: Difference in functional indices is significant in principal component 1 (PC1) between CCB and IPC groups. **B**: Loading plots shows that functional indices including SUR2, eNOS, iNOS activity, PKA activity and eNOS activity mainly account for the difference in PC1. **C**: Difference in structural indices is significant in PC1 between CCB and IPC groups. **D**: Loading plots shows functional indices including VE-cadherin, β-catenin, γ-catenin and P-selectin mainly account for the difference in PC1.

To determine the cardioprotective mechanism of TXL, key target of IPC in cardioprotection was first analyzed using the data from IPC and CCB groups. CCB groups are well separated from IPC (Fig. 7A,C), suggesting that the cardiac structure-function network is disturbed due to different interventions. The scatter points represent the individual's pathophysiological states. Longer distances between the scattered points indicate larger differences of the animal pathophysiological states. The major indices responsible for class separation are revealed by the PCA loading plots (Fig. 7B,D), which represent the importance of all included indices to the inter-group differences within the discrimination model. The PCA loading plots indicate that functional indices (SUR2, eNOS, iNOS activity, PKA activity and eNOS activity) and structural indices (VE-cadherin, β-catenin, γ-catenin and P-selectin) contribute to PC1. As there was a significant difference in PC1 between the CCB and IPC groups, above indices are identified as key targets of IPC in ameliorating IRI.

**Fig 8:**
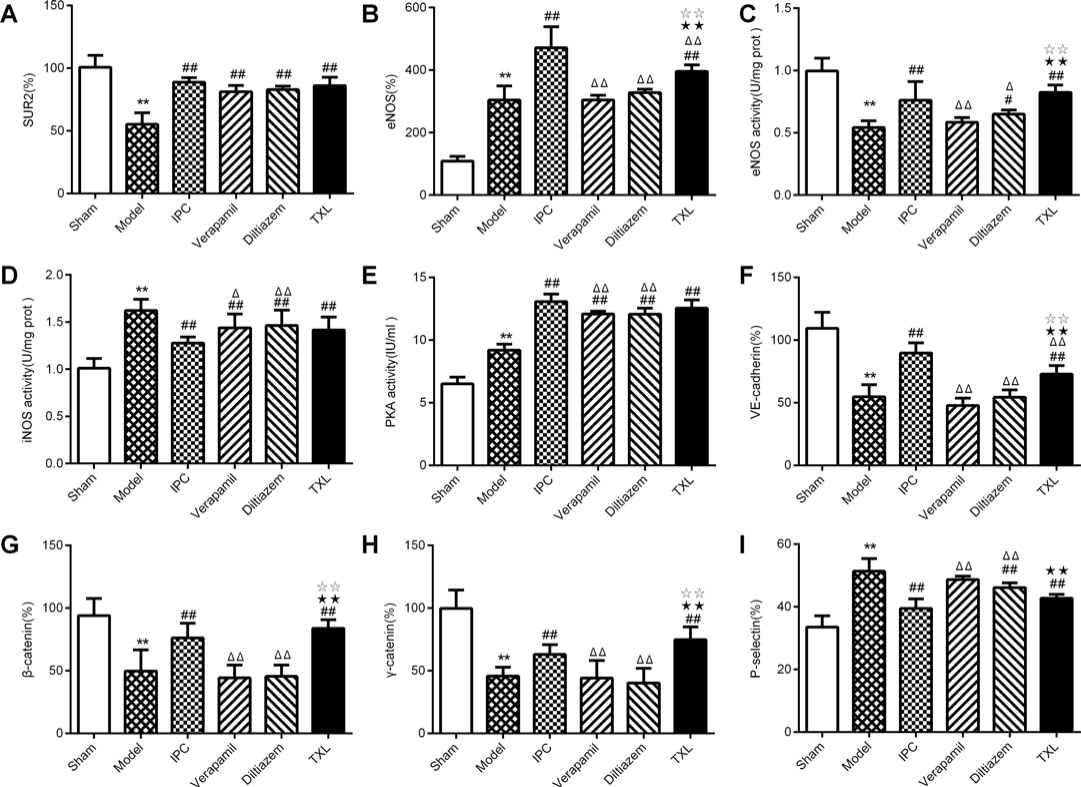
Levels of 9 key indices in different groups. Expression of SUR2, eNOS, VE-cadherin, β-, γ-catenin, P-selectin and activities of eNOS, iNOS and PKA were compared among sham, model, IPC, verapamil, diltiazem and TXL groups. **P<0.01 vs Sham; ^#^ *P*<0.05 vs Model; ^##^ *P*<0.01 vs Model; ^Δ^ *P*<0.05 vs IPC; ^ΔΔ^ *P*<0.01 vs IPC; ^★★^ *P*<0.01 vs verapamil; ^☆☆^ *P*<0.01 vs diltiazem.

To confirm the role of these cardiac structure and function-related targets in mediating the protection of TXL against lethal IRI, the expression of these indices were compared among model, TXL, IPC and CCB groups. Our results show that TXL recovers SUR2, iNOS activity, eNOS activity, VE-cadherin, β-catenin, γ-catenin and P-selectin with a trend toward the sham group, indicating maintaining these indices may induce cardioprotection after reperfusion. Moreover, TXL increases PKA activity and eNOS expression with a trend away from the sham group, indicating activating these indices by TXL may strengthen I/R-induced intrinsic protective signaling pathway, including PKA and eNOS, which is in accordance with our previous studies [5,31]. Among the above nine key indices, eNOS activity, eNOS, VE-cadherin, β-catenin and γ-catenin expression were significantly up-regulated by TXL compared with CCB (all *P*<0.01). Furthermore, there was no significant difference for these five indices between TXL and IPC groups (all *P*>0.05) (Fig. 8). Therefore, these five microvascular barrier-related indices may be the key targets of TXL in minimizing IRI. However, there was no significant difference for SUR2, iNOS activity and PKA activity between TXL and CCB (all *P*>0.05).

## Discussion

To evaluate the severity of lethal IRI, a mathematical model was established based on the relationship between myocardial no-reflow size and necrosis size. IRI severity among different interventions was compared and IPC and CCB groups were identified as the mildest and severest groups, respectively. PCA was applied to further determine 9 key targets of IPC in cardioprotection. Then, microvascular barrier-related indices were confirmed as key targets of TXL in cardioprotection. It is, to our knowledge, the only investigation to address the mechanism of cardioptotection with mathematical modeling and PCA. TXL may prove a useful adjunct in treating diseases with microvascular barrier disruption to alleviate symptoms and prolong survival. Previous study showed that TXL reduced infarct size on day 7 and day 180 after AMI when administered at low loading dose before emergency PCI with aspirin and clopidogrel together [3]. Our study confirmed that pretreatment with TXL could ameliorate lethal IRI, suggesting pretreatment with TXL may improve prognosis in patients at risk of coronary events.

Ischemic heart disease is the leading cause of morbidity and mortality in all industrialized nations. More patients survive arrhythmias with the development of cardiopulmonary resuscitation and timely defibrillation. More patients receive early restoration of coronary flow with Emergency Medical Service transport. Delays in seeking medical help and inherent temporal limitations in initiating thrombolysis or PCI, make it impossible that additional substantive prognosis improvements can be achieved simply via pursuing earlier reperfusion therapy without the development of adjunct therapy [32]. Furthermore, as the outcome is mainly affected by the amount of IRI-induced myocardium loss [33], limiting necrosis size by new adjunctive medicine is imperative to improve prognosis. Recent studies indicated that many factors lead to IRI-induced myocardial necrosis, including microvascular spasm, destruction of microvascular structure, endothelial and myocardial cells swelling, microthrombus and microembolism etc. However, the core mechanism and key approach in cardioprotection was not clarified [33]. With its definite effect in reducing myocardial necrosis size and improving outcome, TXL has been widely used in China for the treatment of patients with coronary artery disease. Our previous studies have provided important mechanistic information, but the entirety of the cardioprotective effects of TXL cannot be explained by a single mechanism. Moreover, TXL has complicated compositions and possible pleiotropic effects and finding the key target of TXL is a compelling, but unfulfilled need. Our study was undertaken to investigate the fundamental mechanism of TXL in minimizing IRI.

Each medicine was administered based on pharmacokinetic character and clinical feasibility. TXL is compound traditional Chinese medicine, so it is impossible to detect the plasma concentration of multiple active constituents of TXL. Therefore, we estimated the time window of its biological effects according to the results of animal experiments. In mini-swine preliminary experiment, TXL reduced infarct size effectively when administered at high loading dose 3-h before ischemia [17] and even at low loading dose 1-h before ischemia [4], suggesting the biological effects of TXL could be reached within 1-h after i.g. delivered. Moreover, there were no other administration routes for TXL except i.g. route, so it was i.g. administered 1-h before coronary occlusion in animal experiment. Diltiazem (peak time = 6-min) and verapamil (peak time = 5-min) were delivered intracoronarily upon reperfusion. Valsartan (peak time = 3-h), rosuvastatin (peak time = 5-h), simvastatin (peak time = 4-h), carvedilol (peak time = 1-h) were administered i.g. at loading dose 1-h before ischemia. Tirofiban (peak time = 30-min, half-life time = 2-h), nicorandil (peak time = 1-h, half-life time = 6-min) and adenosine (half-life time = 6-sec) were delivered as intravenously infusion 1-h before ischemia. Accordingly, to reach their peak bloodstream levels or the biological effects (i.e., TXL) at the time of coronary flow restoration, drugs were administered based on their pharmacokinetic characters and clinical feasibility.

Residual risk still exists after complete coronary restoration and adequate myocardial reperfusion. Two important factors result in development of tissue necrosis, i.e. long-lasting ischemia (no-reflow phenomenon) and lethal IRI. Myocardial ischemia followed by reperfusion results in tissue necrosis termed lethal IRI. Meanwhile, inadequate myocardial perfusion of a given coronary segment without angiographic evidence of mechanical vessel obstruction also results in tissue necrosis termed no-reflow phenomenon [34]. The evaluation method is lacking for lethal IRI, although techniques are available to measure the total extent of infarct size in experimental setting [35]. Accurate determination of infarction resulting from lethal IRI or no-reflow respectively is needed. In our study, severity of lethal IRI was evaluated by pathological staining with or without mathematical modeling respectively. In the model-based pathological approach, NRS was introduced and calculated as follows: NRS = observational necrosis size—estimated necrosis size. Alternatively, in the simple pathological approach, lethal IRI was conceptually calculated as follows: IRI = observational necrosis size—observational no-reflow size. With advantages of mathematical modeling, NRS is with repeatability and accuracy. We confirmed that NRS was positively related to IRI, suggesting NRS could be an alternative marker for evaluation of lethal IRI.

Multi-marker strategy is more informative for pathophysiological evaluation than single marker determination [36–38]. We combined each of the functional markers into PC1, thereby reflecting a general marker of cardiac function. A composite marker of cardiac structure was also introduced. The cardiac structure-function network is confirmed to be strongly associated with NRS. IPC is an effective approach to limit necrosis size by activating the endogenous protective mechanisms of the body. In our study, all interventions were proven to be similarly effective in reducing necrosis size resulting from no-reflow. Surprisingly, we found the NRS is the highest in the CCB group while is the lowest in the IPC group, indicating IPC was more effective than CCB in reducing necrosis size resulting from lethal IRI. Conclusively, we investigated the key target of IPC by analyzing each individual’s physiological fingerprint in IPC and CCB groups.

In our study, we found microvascular barrier function may be the core mechanism in the cardioprotection of IPC. The microvascular endothelial cell monolayer is a semi-permeable barrier that regulates the transendothelial migration of liquid, solutes, plasma proteins and blood cells between the blood and interstitial space [39]. Multiple mediators, along with endothelial cell dysfunction, impair microvascular barrier integrity leading to abnormal microvascular permeability and resultant edema and inflammation [40,41]. Composed of endothelial cells, cell-cell junctions, pericytes and base membrane, endothelial barrier is predominantly regulated by endothelial cell-cell adhesion junction via paracellular pathway [42]. VE-cadherin, β-catenin and γ-catenin are endothelial adhesion junction proteins that cooperate together in maintaining the barrier structure integrity upon inflammatory stimuli [41]. I/R stimulus opens the paracellular pathway to circulating inflammatory mediators, blood cells and water and enlarges necrosis size. Endothelial barrier disruption initiates with agonist—receptor binding, followed by activation of intracellular signaling molecules, including myosin light chain kinase and small Rho-GTPases; these kinases and GTPases then alter the conformation of VE-cadherin, β-catenin and γ-catenin resulting in endothelial hyperpermeability [43]. VE-cadherin is transported from the endothelial cell membrane to the cytoplasm within the first several minutes upon I/R injury [44]. It is possible that IPC prevents the internalization of membrane VE-cadherin, β-catenin and γ-catenin and limits myocardial edema, transendothelial migration, inflammation and consequent necrosis [45–47]. In accordance with a previous study [48], IPC may protect a reperfused heart by enhancing endothelial barrier function.

Our study confirmed that cardioprotection of TXL was related to the key target of IPC, i.e. microvascular barrier function. Previous biological evidence also showed that Rk1, a ginsenoside (ginseng extract, one of active constituents of TXL), could block vascular leakage through actin structure remodeling in retinal endothelial cells [49]. Previous experiments have revealed a decreased expressions of VE-cadherin, β-catenin and γ-catenin after I/R; carvedilol and adenosine instead of CCB could preserve the expression of these junction proteins and reduce necrosis size [13,15,31]. Our study provided a wealth of information on the response of cardiac structure-function network to I/R, which complemented earlier studies in experimental models. Endothelial barrier was disrupted during I/R as described previously[47]. TXL may upregulate VE-cadherin, β-catenin and γ-catenin in a synthesis-dependent manner for hours after I/R injury or prevent the internalization of membrane VE-cadherin, β-catenin and γ-catenin soon after I/R. Conclusively, endothelium may not only be a passive by-stander, but also an active defender against I/R[50]. Our study provided novel evidence on endothelial barrier-dependent mechanism of cardioprotection. As a useful adjunct for treating AMI by endothelial barrier protection, TXL may also be helpful in treating diseases with microvascular barrier disruption, such as diabetic retinopathy[49], proteinuria[51], stoke[52], sepsis[41], and metastatic tumors[53], when confirmed by additional studies.

Apart from endothelial junction proteins, other key targets of IPC were also confirmed, some of which mediated the cardioprotection of TXL. Previous studies have shown that endothelial injury aggravates the inflammatory response through the loss of normal production of eNOS, while induction of eNOS is effective in improving I/R injury and diminishing the injurious effects of oxidative stress. However, iNOS can be induced by cytokines and lipopolysaccharides in the heart and becomes abundant during I/R leading to IRI. In previous studies, increasing the expression and activity of eNOS by activating phosphatidylinositol 3-kinase (PI3K)/protein kinase B (Akt) signaling pathway [54–56] or PKA pathway [23,57] was found to be the main mechanism of IPC against no-reflow. In accordance with previous studies, eNOS activity and expression but not PKA activity was confirmed as the key targets of TXL against lethal IRI in our study. Activating of the K_ATP_ channel is a crucial step in mediating protection of IPC against no-reflow [58–60]. Consistent with a previous study [16], SUR2, a subunit of the K_ATP_ channel, was decreased in reperfused myocardium in our study. Suppressed K_ATP_ channel may reduce mitochondrial permeability, inhibit mitochondrial calcium uptake and increase reactive oxygen species production [61]. We confirmed that IPC instead of TXL protected heart through preservation of K_ATP_. Activated neutrophils accumulate in ischemic myocardium and myocardial inflammation exacerbates after reperfusion. P-selectin translocates upon I/R stimulation from Weibel-Palade bodies in the cytoplasm to the cell membrane. P-selectin relocation mediates the increased leukocyte rolling, leukocyte-endothelial adhesion, leukocyte transmigration across endothelial barrier and resultant aggravated inflammation. In our study, we confirmed that P-selection-mediated interaction between endothelial cells and neutrophils could be partially inhibited by IPC rather than TXL. Accordingly, mechanisms of IPC and TXL in cardioprotection were not exactly the same. Some key targets of IPC mediated the cardioprotection of TXL.

The study has several limitations. Firstly, the mini-swine model of I/R and drug administration, such as drug doses, drug delivery time and route, is only very close to human reality. Secondly, we just selected several representative indices of the network, hoping to be as comprehensive as possible to reflect the network state. Some other endothelial barrier function-related indices could be selected including tight junction proteins, gap junction proteins and focal adhesion proteins. Then it would generate a more complete picture of the relationship between endothelial barrier and cardioprotection. Thirdly, although targets of TXL in cardioprotection have been confirmed in our study. The underlining mechanisms of action were not clear. Further studies are needed to confirm whether TXL targets receptors expressed on the cell membrane or enters into the cells. And studies are also needed to confirm whether it mediates effects through genomic or non-genomic mechanism. Fourthly, our previous study showed that eNOS activity inhibitor Nω-Nitro-L-arginine methyl ester Hydrochloride (L-NAME) partly diminished cardiac protection of TXL against IRI [4]. Inactivation of VE-cadherin gene causes embryonic lethality due to a lack of correct organization and remodeling of the vasculature [62], while VE-cadherin knock-down or VE-cadherin neutralizing antibody[62] may constitute a tool to test the changes of cardiac protection of TXL after reperfusion. Similarly, eNOS, β-catenin and γ-catenin knock out, knock down or inhibitors may be applied to see any changes of TXL cardiac protection against IRI.

## Conclusions

Our study underlines the lethal IRI as one of the causes of myocardial necrosis. Pretreatment with TXL protects against myocardial IRI through enhancing cardiac microvascular endothelial barrier by simulating IPC. Accordingly, pretreatment with TXL could be a valuable adjunct therapy in patients at risk of coronary events to ameliorate lethal IRI.

## Supporting Information

S1 Fig Model diagnostic figure. **A**: Scatterplot of observational necrosis size vs individual estimate **B**: Scatterplot of observational necrosis size vs population estimate, the solid lines indicate the accuracy (diagonal), the dot lines indicate the trends. Both population and individual estimates are associated with observational values. The trend line was close to diagonal, and this model fit the observational values well. **C**: Scatterplot of weight relative standard deviation vs population estimates. **D**: Scatterplot of weight relative standard deviation vs observational necrosis size, the dotted lines is the trend line. The WRSE was relatively well-dispersed between -4 and 4, indicating this model fit the observational necrosis sizes well. Based on the model, 1000 individual estimates of necrosis sizes and 90% (5% and 95% quantile) confidence intervals (CI) were produced as the no-reflow sizes ranged from 0 to 100. Most of the scatter points lied in the yellow strip indicating good predictive ability of this model. **E**: Dispersion of observational values of necrosis and no-reflow sizes in IPC and model group. Observational necrosis sizes from the model group and IPC group were almost evenly distributed in the predicted 90% CI and on both sides of the median lines, suggesting a good capacity of this model to predict necrosis sizes. Abbreviation: WRES = weight relative standard deviation.
(TIF)

S2 Fig Distribution of observational values of necrosis and no-reflow sizes in different groups. **A**: Ischemia postconditioning; **B**: Tongxinluo; **C**: valsartan; **D**: simvastatin; **E**: diltiazem; **F**: verapamil; **G**: carvedilol; **H**: nicorandil; **I**: rosuvastatin; **J**: tirofiban; **K**: adenosine. **L**: Ischemia preconditioning. **M**: Model. Based on the model, 1000 individual estimates of necrosis sizes and 90% (5% and 95% quantile) confidence intervals (CI) were produced as the no-reflow sizes ranged from 0 to 100. Yellow strip shows the 90% CI, dot line is the median line, red scatter is the observational value. Most of the scatter points lied in the yellow strip indicating good predictive ability of this model. The observational values for rosuvastatin, simvastatin and TXL were mainly distributed in the 90% CI. However, the observational values of verapamil and diltiazem were seldom dispersed in the 90% CI.
(TIF)

S3 Fig Lineation of area at risk, area of no-reflow and area of necrosis. Top, the myocardium unstained by Evans blue dye represents the area at risk. Middle, thioflavin S fluorescent dye negatively stained myocardium indicates the area of no-reflow. Bottom, triphenyltetrazolium chloride (TTC)-unstained white myocardium was identified as the area of necrosis.
(TIF)

S1 Materials and Methods Laboratory analyses. (DOC)

S1 Table Estimate of model parameters. (DOC)
