# Supplementary figures and images for: Cardiac Microvascular Barrier Function Mediates the Protection of Tongxinluo against Myocardial Ischemia/Reperfusion Injury
Source: PLoS One. 2015 Mar 17;10(3):e0119846. doi: 10.1371/journal.pone.0119846 (PMC4363146; doi:10.1371/journal.pone.0119846)

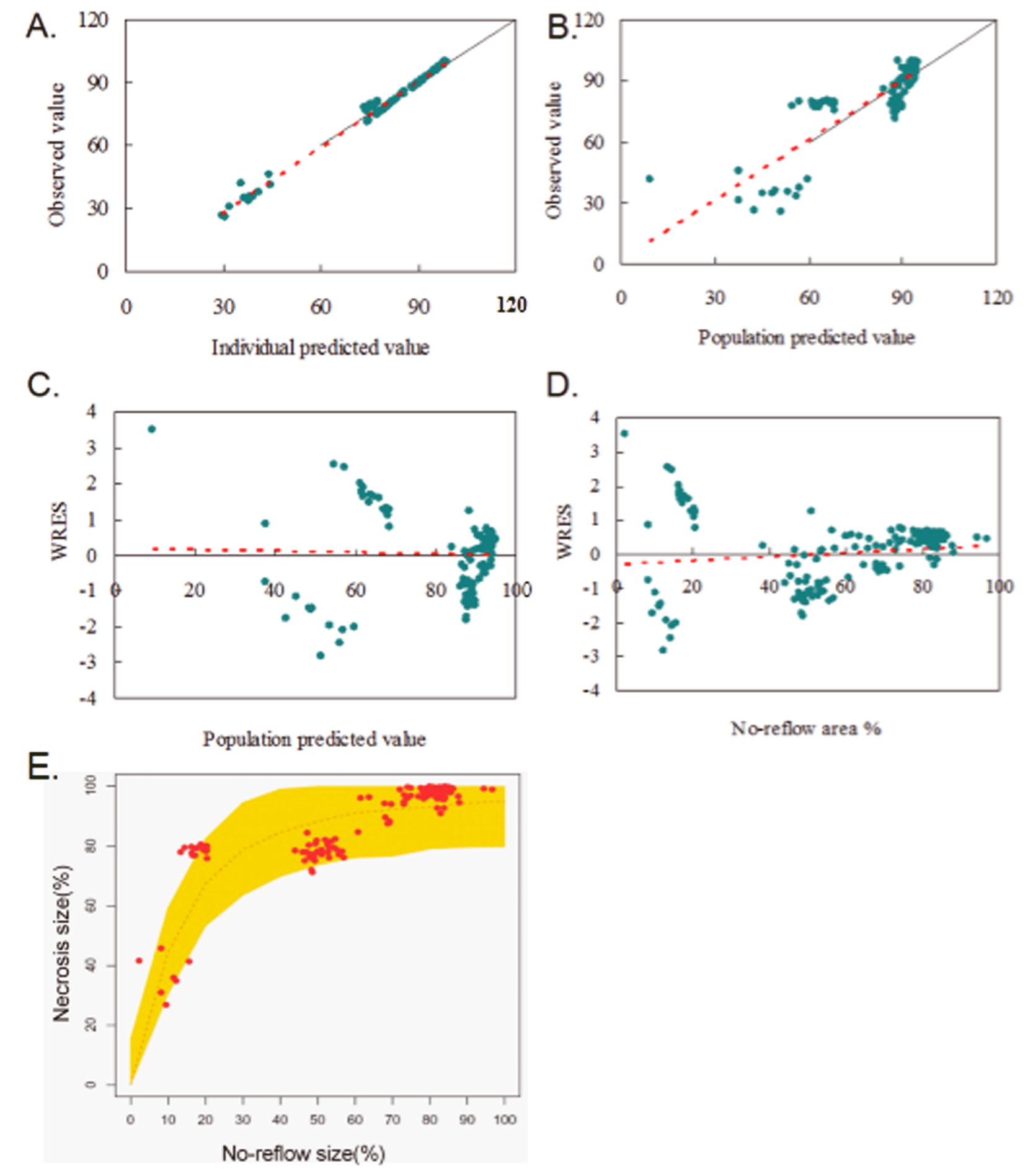

Supplement: S1 Fig — A: Scatterplot of observational necrosis size vs individual estimate B: Scatterplot of observational necrosis size vs population estimate, the solid lines indicate the accuracy (diagonal), the dot lines indicate the trends. Both population and individual estimates are associated with observational values. The trend line was close to diagonal, and this model fit the observational values well. C: Scatterplot of weight relative standard deviation vs population estimates. D: Scatterplot of weight relative standard deviation vs observational necrosis size, the dotted lines is the trend line. The WRSE was relatively well-dispersed between -4 and 4, indicating this model fit the observational necrosis sizes well. Based on the model, 1000 individual estimates of necrosis sizes and 90% (5% and 95% quantile) confidence intervals (CI) were produced as the no-reflow sizes ranged from 0 to 100. Most of the scatter points lied in the yellow strip indicating good predictive ability of this model. E: Dispersion of observational values of necrosis and no-reflow sizes in IPC and model group. Observational necrosis sizes from the model group and IPC group were almost evenly distributed in the predicted 90% CI and on both sides of the median lines, suggesting a good capacity of this model to predict necrosis sizes. Abbreviation: WRES = weight relative standard deviation. (TIF) [file pone.0119846.s001.tif]

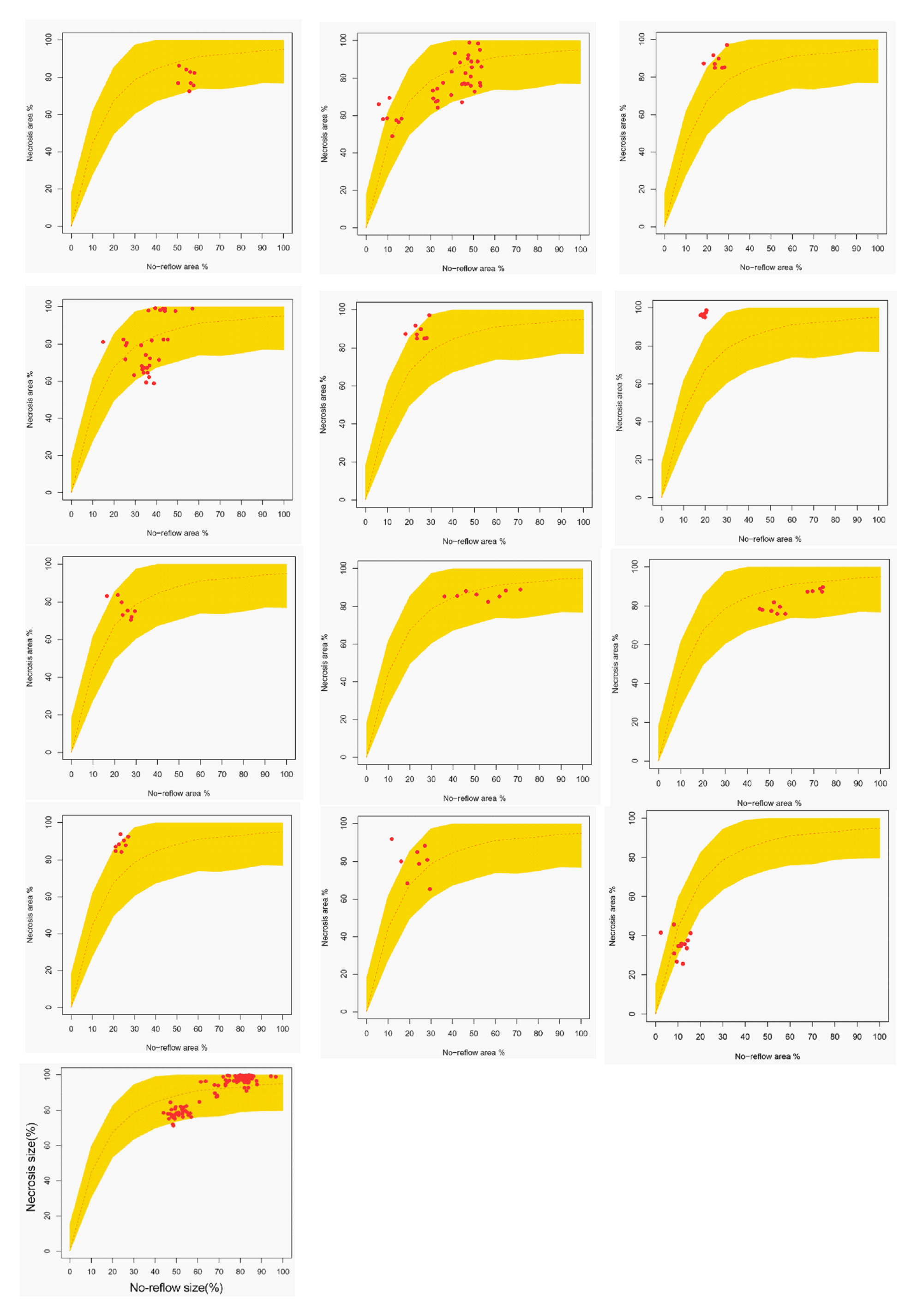

Supplement: S2 Fig — A: Ischemia postconditioning; B: Tongxinluo; C: valsartan; D: simvastatin; E: diltiazem; F: verapamil; G: carvedilol; H: nicorandil; I: rosuvastatin; J: tirofiban; K: adenosine. L: Ischemia preconditioning. M: Model. Based on the model, 1000 individual estimates of necrosis sizes and 90% (5% and 95% quantile) confidence intervals (CI) were produced as the no-reflow sizes ranged from 0 to 100. Yellow strip shows the 90% CI, dot line is the median line, red scatter is the observational value. Most of the scatter points lied in the yellow strip indicating good predictive ability of this model. The observational values for rosuvastatin, simvastatin and TXL were mainly distributed in the 90% CI. However, the observational values of verapamil and diltiazem were seldom dispersed in the 90% CI. (TIF) [file pone.0119846.s002.tif]

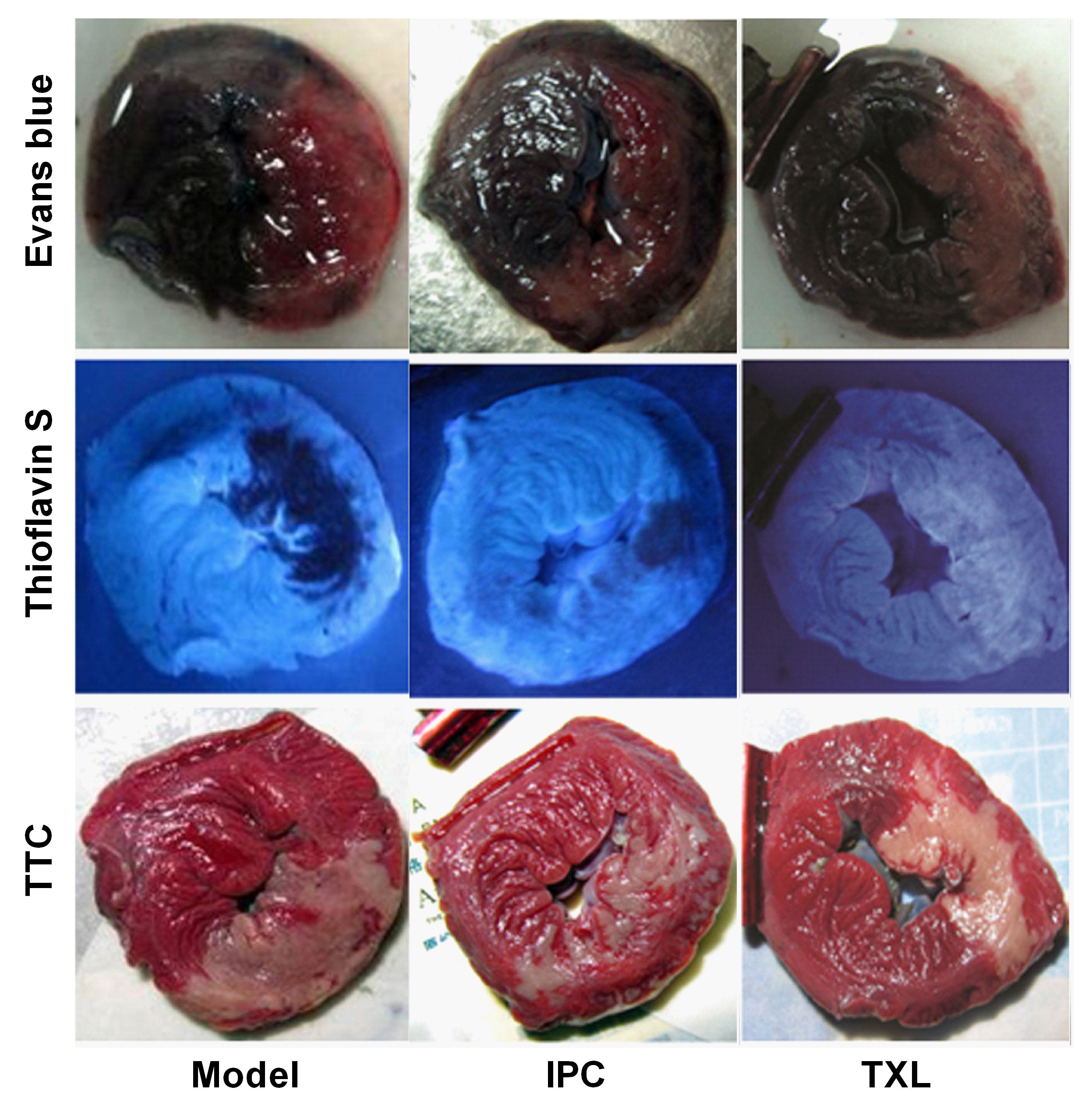

Supplement: S3 Fig — Top, the myocardium unstained by Evans blue dye represents the area at risk. Middle, thioflavin S fluorescent dye negatively stained myocardium indicates the area of no-reflow. Bottom, triphenyltetrazolium chloride (TTC)-unstained white myocardium was identified as the area of necrosis. (TIF) [file pone.0119846.s003.tif]
