# Supplementary material for: Cardiac Microvascular Barrier Function Mediates the Protection of Tongxinluo against Myocardial Ischemia/Reperfusion Injury
Source: PLoS One. 2015 Mar 17;10(3):e0119846. doi: 10.1371/journal.pone.0119846 (PMC4363146; doi:10.1371/journal.pone.0119846)
Supplement: S1 Materials and Methods — (DOC) [file pone.0119846.s004.doc]

**Laboratory analyses**

With the highest and the lowest NRS respectively, calcium channel blockers (CCB) and IPC groups were chosen for laboratory analysis. Meanwhile, TXL group, purpose of this study, and drug-untreated model group were also selected for laboratory analysis. To reveal the full picture of cardiac structure-function networks, 24 markers were measured, such as VE-cadherin, eNOS activity etc. (Table 2) in the Center Laboratory of Fuwai Hospital. Data from sham (n=32), model (n=32), IPC (n=16), verapamil (n=8), diltiazem (n=8) and TXL (n=8) groups were compared.

The expression of eNOS, Ser^1179^p-eNOS, Ser^635^p-eNOS, SUR2, Kir6.1 and Kir6.2, VE-cadherin, β-catenin, γ-catenin, AQP-1, -4, -8, and -9 was detected using Western blot. Equal amounts of protein (30 μg) extracted from myocardial tissues in the area of reflow was applied to the gels. Rabbit polyclonal eNOS (Cell Signaling, USA), rabbit monoclonal p-eNOS (Ser^1179^, Invitrogen, USA), rabbit polyclonal p-eNOS (Ser^635^, Upstate, USA), goat polyclonal SUR2, Kir6.1 and Kir6.2 (Santa Cruz, USA), goat polyclonal VE-cadherin (Santa Cruz, USA), mouse monoclonal β-catenin and γ-catenin (Transduction Labs, USA), rabbit polyclonal AQP-1 (Abcam, UK), rabbit polyclonal AQP-4 (Abcam, UK), rabbit polyclonal AQP-8 (Santa Cruz, USA), goat polyclonal AQP-9 (Santa Cruz, USA), or mouse monoclonal β-actin (Proteintech group, USA) antibodies were applied. The immunoreactive bands were visualized with a chemiluminescence reagent. The intensity ratio of objective band to β-actin corresponded to the relative amounts of objective protein. As previously described, myocardial PKA activity was determined using a nonradioactive PKA assay kit (Promega, USA) . Myocardial activities of iNOS, and cNOS were measured using a spectrophotometrical assay kit (Nanjing JianCheng, China) . Myocardial tissue samples from the area of reflow were homogenized on ice in extraction buffer. The homogenate was centrifuged at 20,000 g for 5 min at 4 °C. The supernatant was assayed for PKA, iNOS and cNOS activity according to the manufacturer's instructions. The activities were reported in units per milligram of myocardial protein. Plasma P-selectin, ICAM-1, VCAM-1, markers of neutrophil-mediated inflammation, were measured using Elisa kits (Rapidbio, USA) after 3-h of reperfusion according to the manufacturer’s instructions.

Neutrophil accumulation in myocardium was evaluated as previously described . Briefly, tissues from area of reflow were embedded in paraffin after fixing in 10% buffered methanal. The segments were cut into 5 µm serial sections before haematoxylin and eosin staining. Neutrophil infiltration was semi-quantified by light microscopy (Nikon eclipse E400, Japan) at 400× magnifications in a blinded manner by a cardiac pathologist. The criterion for identification of neutrophils was a segmented nucleus. The content of neutrophil was graded 0, absent; 1, scant; 2, intravascular plugs; and 3, intravascular plugs and neutrophils present in interstitial space of each section . The results were reported in neutrophils infiltration score per slice of cardiac tissue. Measurement of cardiomyocyte cross-sectional area (CSA), a parameter of cell volume, was performed to determine the amount of cardiomyocyte edema with Leica QWin software. LV coronal sections (5 μm thick) from the middle slice were stained for determining CSA as previously described . To assess CSA, only round to ovoid cells with visible round nucleus were considered, and 50 cells were counted per sample and the average was used for statistical analysis. To quantify mitochondrial edema, mitochondria cross-sectional area (MSA) was measured at a magnification of 30,000× with a JEOL JEM-1230 transmission electron microscope. For comparison of mitochondrial edema, we evaluated electron microscope micrographs of thin sections, and measured the sizes of individual mitochondria. For each experiment, approximately 50 mitochondria were measured in a representative area and the average was used for statistical analysis.

Myocardial water content was determined as reported previously . Briefly, myocardial slices of LV and tissue samples (0.3 g) from the area of reflow were quickly blotted of surface moisture, and weighed. The samples were dried at 80 °C for 48-h, and reweighed. LV and tissue water content was calculated as water content (%) = [(wet weight−dry weight)/ wet weight] ×100%. Myocardial FITC concentration was measured using FITC-dextran and was later quantified using a fluorescence spectrophotometer as previously described . Briefly, 10% FITC-dextran (70 kDa, Sigma, USA) was administered via the tail vein 30-min after reperfusion. Tissue samples (100 mg) from the area of reflow were rinsed, homogenized and cleared by centrifugation. The fluorescent concentration in the supernatant was measured with a fluorescence spectrophotometer via fluorescence at 485 nm excitation/535 nm emission wavelengths.

1. Wu ZQ, Li M, Chen J, Chi ZQ, Liu JG (2006) Involvement of cAMP/cAMP-dependent protein kinase signaling pathway in regulation of Na+,K+-ATPase upon activation of opioid receptors by morphine. Mol Pharmacol 69: 866-876.

2. Li XD, Yang YJ, Geng YJ, Jin C, Hu FH, et al. (2010) Tongxinluo reduces myocardial no-reflow and ischemia-reperfusion injury by stimulating the phosphorylation of eNOS via the PKA pathway. Am J Physiol Heart Circ Physiol 299: H1255-1261.

3. Li XD, Yang YJ, Geng YJ, Cheng YT, Zhang HT, et al. (2013) The cardioprotection of simvastatin in reperfused swine hearts relates to the inhibition of myocardial edema by modulating aquaporins via the PKA pathway. Int J Cardiol 167: 2657-2666.

4. Cheng YT, Yang YJ, Zhang HT, Qian HY, Zhao JL, et al. (2009) Pretreatment with Tongxinluo protects porcine myocardium from ischaemia/reperfusion injury through a nitric oxide related mechanism. Chin Med J (Engl) 122: 1529-1538.

5. Li XD, Cheng YT, Yang YJ, Meng XM, Zhao JL, et al. (2012) PKA-mediated eNOS phosphorylation in the protection of ischemic preconditioning against no-reflow. Microvasc Res 84: 44-54.

6. Barrabes JA, Garcia-Dorado D, Gonzalez MA, Ruiz-Meana M, Solares J, et al. (1998) Regional expansion during myocardial ischemia predicts ventricular fibrillation and coronary reocclusion. Am J Physiol 274: H1767-1775.

7. Garciarena CD, Caldiz CI, Portiansky EL, Chiappe de Cingolani GE, Ennis IL (2009) Chronic NHE-1 blockade induces an antiapoptotic effect in the hypertrophied heart. J Appl Physiol (1985) 106: 1325-1331.

8. Ikeno F, Inagaki K, Rezaee M, Mochly-Rosen D (2007) Impaired perfusion after myocardial infarction is due to reperfusion-induced deltaPKC-mediated myocardial damage. Cardiovasc Res 73: 699-709.
