# Supplementary material for: Cardiac Microvascular Barrier Function Mediates the Protection of Tongxinluo against Myocardial Ischemia/Reperfusion Injury
Source: PLoS One. 2015 Mar 17;10(3):e0119846. doi: 10.1371/journal.pone.0119846 (PMC4363146; doi:10.1371/journal.pone.0119846)
Supplement: S1 Table — (DOC) [file pone.0119846.s005.doc]

**Table S1 Estimate** of model parameters

| Parameters | Value | RSE(%) |
| --- | --- | --- |
| EC_50_,% | 11.7 | 10.7 |
| r | 1.39 | 7.8 |
| ISV, % | 8.24 | 12.9 |
| ε, % | 3.99 | 12.4 |

EC_50_ is the no-reflow size when E_AN_ is equal to half of the largest necrosis size(E_max_=50), r is the slope factor, ISV is the variation among trials, and ε is the individual residual. RSE=relative standard deviation.
